# Vitamin D - roles in women's reproductive health?

**DOI:** 10.1186/1477-7827-9-146

**Published:** 2011-11-02

**Authors:** Magdalena Grundmann, Frauke von Versen-Höynck

**Affiliations:** 1 Department of Obstetrics, Gynecology and Reproductive Medicine, Hannover Medical School, Carl-Neuberg-Strasse 1, 30625 Hannover, Germany

**Keywords:** vitamin D, women's health, reproduction, fertility, pregnancy

## Abstract

In the past few years a growing interest in vitamin D can be observed in the lay and biomedical literature due to findings demonstrating a low vitamin D status in the population. In addition to its importance for the regulation of calcium and phosphorus homeostasis recent epidemiologic studies have observed relationships between low vitamin D levels and multiple disease states. This secosteroid hormone also regulates the expression of a large number of genes in reproductive tissues implicating a role for vitamin D in female reproduction. In this report we summarize the recent evidence that vitamin D status influences female reproductive and pregnancy outcomes. Human and animal data suggest that low vitamin D status is associated with impaired fertility, endometriosis and polycystic ovary syndrome. Evidence from observational studies shows higher rates of preeclampsia, preterm birth, bacterial vaginosis and gestational diabetes in women with low vitamin D levels. However, confirmation of experimental observations establishing an association of vitamin D deficiency with adverse reproductive outcomes by high quality observational and large-scale randomized clinical trials is still lacking. The determination of optimal 25(OH)D3 levels in the reproductive period and the amount of vitamin D supplementation required to achieve those levels for the numerous actions of vitamin D throughout a woman's life would have important public health implications.

## Background

**Figure 1:**
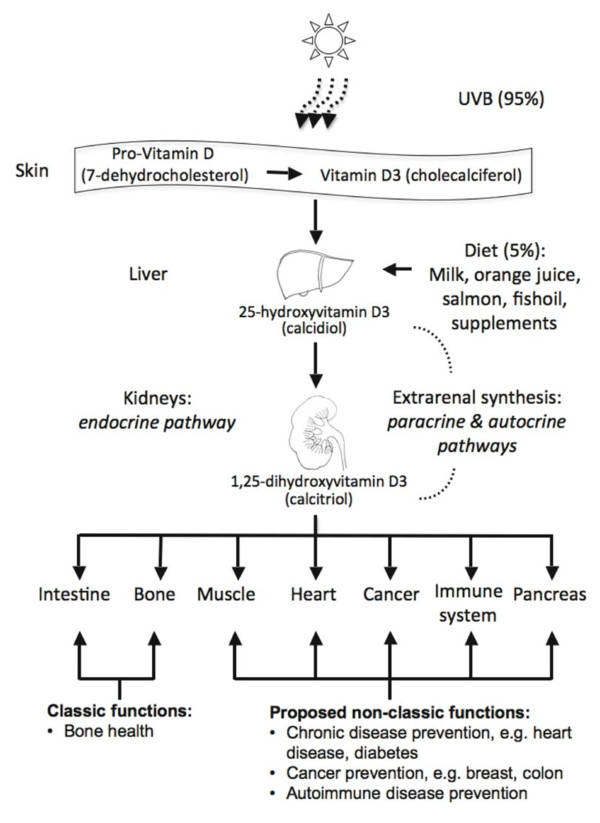
**Types and functions of vitamin D**.

In North America and Western Europe only small amounts of vitamin D, a fat-soluble secosteroid hormone, enter the metabolic circle via dietary uptake (e.g. from fish), although dietary supplement intake has increased in recent years (Figure 1). The main source (about 95%) is vitamin D_3 _(cholecalciferol) that is photochemically synthesized in the skin by ultraviolet-B radiation. Thermal conversion of pro-vitamin D_3 _(7-dehydrocholesterol) leads to pre-vitamin D_3_, which isomerizes into cholecalciferol. Cholecalciferol is bound to serum vitamin D-binding protein (DBP) and through a two-step enzymatic pathway involving 25-hydroxylase of the liver and 1α-hydroxylase (CYP27B1) of the kidney and extrarenal tissues, it is converted to the biologically active hormone calcitriol (1α,25(OH)_2_D_3_) [1]. In a wide variety of cell types vitamin D exerts its effects by binding to the vitamin D receptor (VDR), a member of the nuclear steroid receptor superfamily and an intracellular transcription factor [2]. The regulation of VDR expression is one of the main mechanisms through which target cells respond to calcitriol so that polymorphisms of this receptor can change the usual mode of functioning.

Although 1,25(OH)_2_D_3 _is the biologically active form of vitamin D the vitamin D status is mainly assessed by the measurement of serum 25(OH)D_3 _concentration. The test is used due to associations between vitamin D, calcium and plasma parathyroid hormone (PTH) [3,4]. While becoming vitamin D deficient the parathyroid glands increase the secretion of PTH to accelerate renal calcium reabsorption and calcium release from bone. Another effect of PTH is the stimulation of renal 1,25(OH)_2_D_3 _production with possibly misleading 1,25(OH)_2_D_3 _concentrations within the normal range.

**Figure 2:**
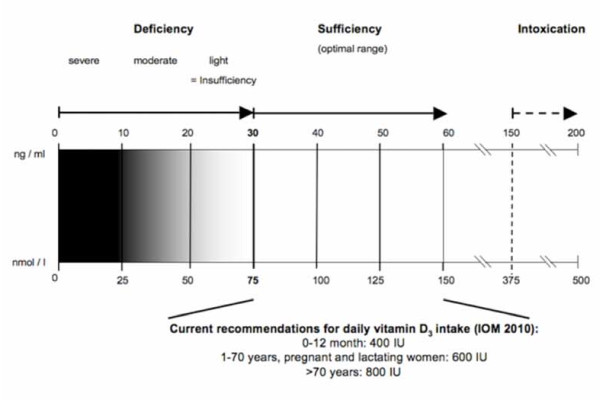
**Classification of vitamin D status and recommendations for substitution**.

Based on associations between plasma 25(OH)D_3 _and PTH concentration, calcium absorption, bone turnover markers, and bone mineral density, investigators have argued that a plasma 25(OH)D_3 _concentration of greater than 75 nmol/l (to convert to ng/ml divide by 2.5) is appropriate to define vitamin D sufficiency [5-7]. Precisely defining vitamin D deficiency or insufficiency is still a matter of great debate. A rather simplistic scheme for the classification of vitamin D status is shown in Figure 2. Although there is currently no consensus for optimal levels, most experts use a plasma concentration of 25(OH)D_3 _of 25 nmol/l as a conventional cut-off for defining the lower limit of adequacy of vitamin D status [8]. These cutoffs were derived from populations of non-pregnant individuals. However, recent evidence demonstrates that the prevalence of vitamin D deficiency in women of childbearing age is surprisingly high [9-12].

**Figure 3:**
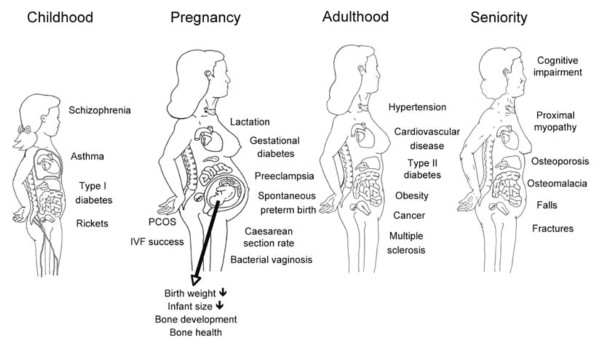
**Proposed health implications of vitamin D deficiency from infancy to seniority**.

Vitamin D plays a pivotal role in bone metabolism and mineral homeostasis. Emerging data identify critical roles for the active form of vitamin D (1α,25(OH)_2_D_3_) in a variety of other biological processes including regulation of cellular growth, differentiation and metabolic modulations [13,14]. Beneficial roles for vitamin D in a spectrum of pathologic processes, including autoimmunity, insulin resistance, cardiovascular disease, and malignancies, are emerging concomitantly with the appreciation of a global pandemic of vitamin D deficiency (Figure 3) [5,13,14]. Over the past decade the physiological role of 1α,25(OH)_2_D_3 _has been investigated extensively [15], but data regarding its role in human reproduction are scarce [16-18]. Since the VDR and 1α-hydroxylase are expressed in reproductive tissues including ovary, uterus, placenta, testis and hypophysis an association with vitamin D and many potential pathways linking vitamin D to reproductive health outcomes almost suggests itself [19-21].

The aims of this review are to critically summarize the most recent data regarding the association of vitamin D deficiency and female reproductive outcomes. We searched the PubMed and MEDLINE up to August 20 2011 using combinations of the following keywords: vitamin D deficiency/insufficiency and pregnancy, reproduction, fertility/infertility, polycystic ovary syndrome, endometriosis, preeclampsia, gestational diabetes, preterm birth and bacterial vaginosis. Randomized controlled trials, original papers and review articles are included in the present article.

### Health implications of vitamin D deficiency in female reproduction

#### Vitamin D and fertility

A seasonal distribution in human natural conception and birth rates has been consistently demonstrated, showing a peak conception rate during summer in northern countries with strong seasonal contrast in luminosity [22]. Experimental studies have demonstrated that the ovary is a target organ for 1,25(OH)_2_D_3 _raising the possibility that this active metabolite of vitamin D_3 _might play a role in modulating ovarian activity [23]. Experiments investigating the significance for fertility and reproductive capacity, demonstrate that 25(OH)D_3_-deficient female rats had reduced fertility rates, decreased litter sizes and compromised mating behavior [24]. VDR-null mice showed hypergonadotropic hypogonadism with decreased aromatase activities in the ovary, testis and epididymis, uterine hypoplasia, impaired folliculogenesis; decreased sperm counts, decreased sperm motility and histological abnormalities of the testis [19]. Ablation of 1α-hydroxylase in female mice is accompanied by abnormal ovarian follicle development, uterine hypoplasia and infertility similar to VDR knockout mice [25,26]. However, when serum calcium and phosphorus were normalized by a rescue diet in the female 1α-hydroxylase -/- mice, the defective phenotype including dysfunction in the hypothalamic-pituitary-ovarian axis and ovarian angiogenesis were reversed. The authors conclude that the infertility seen in 1,25(OH)_2_D_3_-deficient mice is an indirect effect mediated by extracellular calcium and phosphorus and not a direct effect of vitamin D deficiency [27,28]. Specific human data in this context is sparse. The first study looking at vitamin D and in vitro-fertilization (IVF) success in 10 healthy women undergoing IVF and embryo transfer found an association of raised oestradiol levels during gonadotrophin-induced ovarian stimulation and a significant increase of serum 1,25(OH)_2_D_3_. (r = 0.787, p < 0.001) [29,30]. Ozkan et al. recently showed higher pregnancy and implantation rates across tertiles of 25(OH)D_3 _in follicular fluid of 84 infertile women undergoing IVF and proposed follicular fluid 25(OH)D_3 _levels as an independent predictor to success of an IVF-cycle [31] whereas two prospective studies with 101 and 82 women could not confirm these findings [32,33]. On the contrary no significant differences in pregnancy rates and embryo quality were found between patients with low (< 50 nmol/l) and moderate (50-75 nmol/) 25(OH)D_3 _follicular fluid levels, at high vitamin D levels in follicular fluid (> 75 nmol/l) even a decrease in pregnancy rate and embryo quality was seen [32,33]. It was also shown that increased follicular fluid 25(OH)D_3 _levels in combination with decreased follicular fluid glucose levels have a negative impact on embryo quality and therefore on IVF outcome [33]. Moreover, Estes and colleagues found a decreased expression of DBP in the follicular fluid of the IVF success group [34]. While the results of human studies are contradictory the role of vitamin D on human fertility and reproductive physiology merits further assessment by appropriate longitudinal studies.

#### Vitamin D and polycystic ovary syndrome

Polycystic ovary syndrome (PCOS) is among the most common endocrine disorders in women of reproductive age and has a strong genetic component. It is characterized by ovarian dysfunction and its clinical manifestations may include obesity, increased insulin resistance and compensatory hyperinsulinemia, oligo-/anovulation and infertility [35]. Studies regarding vitamin D status in patients with PCOS show an inverse correlation between vitamin D levels and metabolic risk factors, e.g. insulin resistance, BMI, waist-to-hip-ratio, triglycerides, total testosterone and DHEAS and a positive correlation with insulin sensitivity [36-38]. Data on the role of gene variants involved in vitamin D metabolism in PCOS are sparse but suggest an association of VDR and vitamin D level-related variants with metabolic and endocrine parameters in women with PCOS [39]. Several studies although limited by modest sample sizes have suggested associations between VDR polymorphisms and the development of PCOS as well as insulin resistance [39-43]. Different distributions e.g. of VDR Apa-I and Fok-I gene polymorphisms were found in a cohort of 162 women with PCOS and their controls [42]. It seems possible that variants in the VDR through their effect on luteinizing hormone, sex hormone binding globulin levels and testosterone are involved in the pathogenesis of PCOS [39,40]. Further genes involved in vitamin D synthesis, hydroxylation and transport and their role in PCOS are currently under investigation [39].

Clinical trials with either vitamin D supplementation or administration of vitamin D_3 _analogues showed positive effects on insulin secretion, lipid profile, menstrual cycle and follicular development and a decrease of fasting and stimulated glucose and C-peptide levels [44-48]. However, most of the studies had rather small sample sizes and experimental set-up was quite heterogenous. One of the great confounders in all studies was the presence of obesity. In some studies an association of vitamin D and insulin resistance was only seen in obese patients or 25(OH)D_3 _levels were only associated with obesity and insulin resistance but not with PCOS per se [36,49,50]. Lower serum levels of 25(OH)D_3 _were shown in obese PCOS patients (e.g. 31.9 ± 9.4 nmol/l) than in non-obese (73.1 ± 20.2 nmol/l) [36,51]. Consequently, the association of hypovitaminosis D with features of PCOS may be associated with obesity but not with the presence of PCOS. Recently it was hypothesized that vitamin D deficiency is not only in association with obesity but a potentially reason [52]. As vitamin D supplementation evidently has positive effects on the outcome of PCOS the question whether to substitute patients with PCOS to ameliorate insulin resistance and prevent other health complications, such as diabetes mellitus type 2, has to be addressed in large intervention trials.

#### Vitamin D and endometriosis

An impairment of immunologic mechanism and inflammatory responses has been suggested to be involved in the pathogenesis of endometriosis. Cyclic and early pregnant endometrium is an extrarenal site of vitamin D synthesis and action [21]. In endometriosis patients the gene encoding for 1α-hydroxylase shows an enhanced expression in ectopic endometrium [21]. Recent data also indicate that women suffering from endometriosis express more VDRs in their endometrial tissue [53] and have higher serum levels of 25(OH)D_3 _than those of the control group [54,55]. An analysis of VDR gene polymorphisms (ApaI, TaqI, FokI, BmsI) including 132 women with endometriosis and 133 controls found relatively similar VDR polymorphism genotype frequencies in cases and controls [56]. A small observational study in 26 women with and 17 women without endometriosis detected insignificantly higher serum and lower peritoneal DBP concentrations [57]. However, Faserl and colleagues found about 3 times higher DBP levels associated with the GC*2 allele product that was in greater concentration in serum pools, as well as in single validation samples in 56 women with endometriosis compared to healthy controls. This finding may be explained by an influence of vitamin D on local activity of immune cells and cytokines maintaining endometriosis and an insufficiency to activate macrophage's phagocytotic function in those carrying the GC*2 polymorphism [58,59].

#### Vitamin D and hypertensive disorders of pregnancy

Hypertensive disorders of pregnancy and especially preeclampsia (PE) are the most studied reproductive health outcomes in association with maternal vitamin D status. The syndrome PE is defined as the occurrence of hypertension and proteinuria after 20 weeks of gestation, and with a prevalence of 3-5% of all pregnancies worldwide it is the leading cause of maternal and fetal morbidity and mortality [60]. Seasonal patterns in PE suggest a role for vitamin D and sunlight, because of a higher incidence in winter and a lower incidence in summer [61,62]. Compared with normal pregnancies, PE is characterized by marked changes in vitamin D and calcium metabolism [63] and already in the early 1990's a role for vitamin D in the pathogenesis of PE was hypothesized [64]. Women with PE are known to have lower circulating 25(OH)D_3 _levels than normotensive pregnant women [65-68].

In nested case-control studies vitamin D deficiency in pregnancy < 50 nmol/l of 25(OH)D_3 _was associated with an almost 4-fold odds of severe PE [69] and vitamin D deficiency < 37.5 nmol/l was even associated with a 5-fold risk of developing PE [65]. Bodnar et al. showed that 25(OH)D_3 _deficiency before 22 weeks of gestation is an independent risk factor for the manifestation of PE [65]. Interestingly, Robinson and colleagues recently reported lower maternal 25(OH)D_3 _concentrations in 56 women with early onset PE and small for gestational age (SGA) infants vs. infants with normal fetal growth suggesting an impact of vitamin D on fetal growth through placental mechanisms [70]. In a case-control study in 78 women near term with at least two blood pressure readings above 140/90 mmHg and 109 controls, women with low circulating 25(OH)D_3 _concentrations were more likely to have hypertension [71]. In an earlier study hypertensive pregnant women were not different from the normotensive ones regarding plasma corrected calcium and phosphate and urinary excretion of calcium and phosphate, but had lower total and free calcitriol index (ratio of total calcitriol on DBP) [72].

Not all of the data regarding vitamin D status and PE prevalence are consistent and studies reporting no relationship have significantly lower case numbers [73-75].

While studies in the second and third trimester of pregnancy suggest and association of hypovitaminosis D and PE risk a nested-case control study conducted by Powe et al. in first trimester found that total and free 25(OH)D_3 _levels were not independently associated with subsequent PE [75] and a recent prospective cohort study of 221 Canadian women with high risk of PE found no difference in the rates of PE, gestational hypertension, preterm birth or composite adverse pregnancy outcomes by 25(OH)D_3 _concentration [74]. The cited studies on vitamin D and PE are not only conducted in different populations but also differ considerably in their experimental set-up, definition of vitamin D deficiency, inclusion criteria and possible confounders so that the discrepancy in study results can not easily be explained and large-scale clinical trials are awaited to clarify this issue. However, vitamin D supplementation studies to prevent PE showed protective effects of vitamin D. The first known study in this context was a controlled trial in London in the 1940's -1950's with 5644 women in which a reduction of 31.5% in PE was seen in women who received a dietary supplement containing vitamins (2,500 IU vitamin D), minerals and fish oil in comparison to the control group who did not receive any supplement [76]. A recent study by Haugen et al. in a cohort of 23,423 nulliparous women in Norway showed a 27% reduction in the risk of PE in women who took 400-600 IU vitamin D supplements per day compared to women without supplementation [77]. One small RCT conducted in India, however finds no association of vitamin D supplementation (1,200 IU vitamin D/day and 375 mg calcium/day) with a reduced risk for PE but a reduction in diastolic blood pressure of 8 mmHg [78]. In a unique investigation in a Finish birth cohort, Hypponen et al. observed that vitamin D supplementation early in the first year of life is associated with a 50% reduction of PE prevalence in the first pregnancy later in life [79]. This suggests that vitamin D intake in infancy may be involved in programming processes of the immune system.

It remains unclear how vitamin D insufficiency or deficiency might be involved in pathophysiologic processes that cause PE but it may be linked with PE through actions outside of its traditional calcium regulatory role. One mechanism suggested is the regulation of maternal and placental immunological and inflammatory responses, as it has been shown in experimental models [80,81]. The placenta itself expresses 1α-hydroxylase and thus produces the active metabolite 1α,25(OH)_2_D_3 _[82]. Whether this placental production of vitamin D is a major contributor to the maternal vitamin D status or has mainly paracrine functions is controversial [83,84]. In syncytiotrophoblasts from preeclamptic pregnancies the expression and activity of 1α-hydroxylase are restricted suggesting an important role for vitamin D at the placental site of the disease [85]. There is evidence that vitamin D regulates key target genes associated with implantation, trophoblast invasion and implantation tolerance [86]. Regarding implantation tolerance, Th2 cell induction is one of the critical steps required for the maintenance of normal pregnancy whereas impaired implantation and adverse reaction of maternal metabolism to the fetus in PE is mediated by Th1-cytokines [87]. This fetal placental interface may be influenced by vitamin D, which has an important role in promoting the shift to a Th2-dominated immune response pattern [88]. The maternal response to reduced placental perfusion in PE may equally be affected by vitamin D. Maternal vitamin D deficiency may lead to the increased inflammatory response that characterizes PE as well as to endothelial dysfunction through direct effects on angiogenesis gene transcription, including vascular endothelial growth factor (VEGF) [89,90].

#### Vitamin D and gestational diabetes mellitus

Gestational diabetes (GDM) is becoming increasingly more common and has long-term implications for the health of mothers and their children. The former have an increased risk of developing type 2 diabetes, while their offspring have an increased risk of obesity and diabetes later in life [91]. Polymorphisms of vitamin D have been associated with metabolic mechanisms, e.g. insulin release and the maintenance of glucose tolerance [92]. A genetic contribution of CYP27B1 polymorphisms may modulate 25(OH)D_3 _levels in GDM patients as reported by Ramos-Lopez et al. [93]. GDM in relation to maternal vitamin D status is examined only in observational studies [94-100]. In a nested case-control study 25(OH)D_3 _levels < 50 nmol/l at 16 weeks gestation before the onset of GDM was associated with a 2.7-fold increased risk for the development of GDM later in pregnancy independent of measured confounders [96]. At the time of oral glucose tolerance testing at mid gestation two reports noted significantly positive correlations between 25(OH)D_3 _concentrations and insulin sensitivity [94,98] or fasting/2-hour blood glucose levels and HbA(1c) [100] whereas no association was observed in another large study in South India [99]. In another recent study the prevalence of severe vitamin D deficiency (25(OH)D_3 _< 37.5 nmol/l) in the second trimester of pregnancy was higher in GDM compared to normoglycemic pregnant women [95].

Interestingly, after 1,25(OH)_2_D_3 _supplementation a decrease in glucose and insulin levels was noted [101]. Lau and colleagues contribute to this evidence by demonstrating that, in women with GDM a lower serum 25(OH)D_3 _concentration was independently associated with poorer glycemic control [100]. RCTs of vitamin D supplementation, initiated early in pregnancy, are now required to demonstrate whether vitamin D supplementation might reduce the incidence or severity of GDM.

#### Vitamin D and mode of delivery

Data from Merewood et al. show an inverse association with having a primary cesarean section and vitamin D status in 253 women. Severely vitamin D deficient women with levels of 25(OH)D_3 _< 37.5 nmol/l delivered nearly four times as often by cesarean section than those with 37.5 nmol/l or greater (OR 3.84) [102]. No association was found between obstructed labor ensuing cesarean section and vitamin D status in a case-control study (37 cases, 80 controls) of nulliparous women at term in Pakistan [103]. These are the only studies so far examining vitamin D status in pregnancy and mode of delivery and therefore the issue needs further investigation.

#### Vitamin D and spontaneous preterm birth

Spontaneous preterm birth (SPB) occurs before 37 weeks gestation. From a pathophysiological point of view there are numerous reasons for SPB including intrauterine infection and inflammation. One major factor is the presence of bacterial vaginosis, a disruption of the normal balance of vaginal flora with increased growth of anaerobic bacteria responsible for the release of inflammatory cytokines, prostaglandins, and phospholipase A_2 _[104-106]. A recent study by Bodnar et al. showed a linear inverse dose-response association between maternal vitamin D status and the prevalence of bacterial vaginosis in early pregnancy [107]. Using a large, representative data set with inclusion of 3,523 women Hensel et al confirmed the association of 25(OH)D_3 _deficiency (< 75 nmol/l) and bacterial vaginosis among pregnant women [108]. In a cross-sectional study of African American adolescents bacterial vaginosis was shown to also be associated with vitamin D insufficiency (25(OH)D_3 _< 50 nmol/l) [109].

Since vitamin D has immunmodulatory and anti-inflammatory effects, such as the regulation of production and function of cytokines and neutrophil degranulation products that is important and relevant to prevent microbial invasion one may expect a protective effect on SPB risk [110-112]. The various cells of the immune system express VDRs and are modulated by vitamin D [113]. Although vitamin D action dampens the activation of the acquired immune system in response to autoimmunity, this hormone has key actions that enhance the innate immune system. It is involved in cell-mediated immunity by reducing the production of inflammatory cytokines such as IL-1, 6 and TNF α that are involved in SPB [89,110,114,115]. Human decidual cells are able to synthesize active 1,25(OH)_2_D_3_. Therefore several studies point to the fact that vitamin D is involved in the regulation of acquired and innate immune responses at the fetal-maternal interface across gestation [89,110,116]. Vitamin D might reduce the risk of SPB also by helping to maintain myometrial quiescence. Myometrial contractility is dependent on calcium release within the muscle cell and this process is regulated by vitamin D [117,118]. Although experimental data are promising there is limited observational data available on the relationship of vitamin D and SPB. A recent prospective cohort study in 221 Canadian women could not show an association of 25(OH)D_3 _deficiency (< 50 nmol/l) or insufficiency (< 75 nmol/l) with preterm birth [74] whereas in another observational study with 14 cases maternal 25(OH)D_3 _levels of < 28 nmol/l at 28 to 32 weeks of gestation were associated with a 0.7 weeks shorter gestation in an all Caucasian sample but not at 11 weeks gestation [119]. In another first trimester cohort of 4,225 women with 40 cases of SPB ≤34 6/7 weeks the prevalence of vitamin D deficiency (25(OH)D_3 _< 50 nmol/l) was comparable among women who subsequently delivered preterm compared with controls [120]. 25(OH)D_3 _levels < 80 nmol/l in HIV-positive pregnant Tanzanian women were not associated with an increased risk for preterm delivery [121]. In a cohort of 82,213 singleton live births Bodnar et al. found indirect evidence that vitamin D and seasonal sunlight exposure are relevant for preterm birth. The prevalence of SPB was lowest among women who conceived in summer and fall and was highest among winter and spring conceptions [61,122]. The strongest evidence that vitamin D sufficiency may protect against preterm birth may be reported in the near future by Hollis and colleagues from a randomized controlled trial of 600 white, black, and Hispanic mothers and supplementation of 400 IU, 2,000 IU, or 4,000 IU/d of vitamin D_3 _in early pregnancy [123]. More large studies are awaited to validate these important findings that might represent vitamin D supplementation as a simple and inexpensive method to reduce the risk of this adverse pregnancy outcome.

#### Vitamin D and other reproductive outcomes

Vitamin D seems to have a complex relation with fetal growth that may vary by genotype, race and other variables that could not be identified yet. This relation between maternal vitamin D status and fetal birth weight has been studied in RCT's [124-127] and a number of observational studies [15,99,119,128-135] with mixed results. Due to confounding factors (e.g. maternal nutritional status, calcium and phosphorus intake, maternal prepregnant BMI, socioeconomic status), that were not always considered in these analyses the outcomes are hard to compare.

Despite the recognized role of vitamin D in bone metabolism and prevention of osteoporosis in postmenopausal women, the information available on the impact of vitamin D status on maternal and fetal bone health is limited. No systemic studies have examined the effect of vitamin D status in the neonate on skeletal mineral content in humans, particularly studies regarding isolated vitamin D effects clearly separated from calcium effects are very rare. Only a handful of observational studies [119,128,131,136-139] and one RCT [124] reported contradictory results. Therefore the importance of maternal vitamin D status to fetal skeletal development is not sufficiently investigated. Nevertheless we would like to point the interested reader to well-written reviews of the above-mentioned topics [16,140,141].

Breast milk is an ideal nutrient for a newborn but is not sufficient to maintain newborn vitamin D levels within a normal range. Many nursing mothers or their infants require vitamin D supplementation for optimal health. Because of the existence of well-written reviews analyzing the available literature on vitamin D and lactation we refer the reader to these articles [140,142-144].

#### Vitamin D and fetal programming

Vitamin D induces more than 3,000 genes, many of which have a role in fetal development [145]. Therefore, vitamin D may be particular relevant to the "developmental origins" or "fetal programming hypothesis" in which environmental factors such as vitamin D influence the genomic programming of fetal and neonatal developmental and subsequent disease risk in both childhood and adult life [146]. Interestingly, in later life, children of mothers with low vitamin D serum levels during pregnancy suffer more often from chronic diseases such as wheezing and asthma [147-149], schizophrenia [150,151], multiple sclerosis [152], type 1 diabetes mellitus and insulin resistance [153-158] suggesting intrauterine programming as possible mechanism [17,143,159,160]. Mechanisms underlying this long-term effect of the intrauterine environment are not known yet but epigenetic mechanisms that lead to persistent changes in structure and function in endocrine systems are hypothesized.

#### Vitamin D supplementation and toxicity issues

Although summer sunlight is the most potent source of vitamin D, fortified foods or specific supplements take on an increasing importance, especially in the winter at northern latitudes (40°N). Dietary recommendations are available in many European countries, the US and also globally [161,162]. The updated dietary reference intakes for calcium and vitamin D of the IOM published for the US and Canada (e.g. IOM 600 IU for pregnant/lactating women) are based primarily on the intake of vitamin D to ensure skeletal health (Figure 2). Levels of 25(OH)D_3 _of 75-110 nmol/l provided optimal benefits for these outcomes and can be obtained with daily doses of 1,800-4,000 IU [163,164]. Concerning optimal concentrations of 25(OH)D_3 _the IOM link serum 25(OH)D_3 _levels ≥50 nmol/l with the Recommended Dietary Allowance that meets 97,5% of the US population's needs and a level of < 30 nmol/l with increased risk for vitamin D-deficient rickets [162]. Supraphysiologic, potentially toxic levels are defined as a 25(OH)D_3 _concentration above 150 nmol/l [5].

The optimal serum levels and intake for pregnancy are not known [160,165]. While low doses of 400, 800 and 1,600 IU did little to improve vitamin D nutritional status in pregnant women [126] in a very recent randomized controlled trial vitamin D supplementation of 4,000 IU/d started at 12-16 weeks of gestation was safe and most effective achieving sufficient levels in women and their neonates regardless of race [166]. As with other micronutrients, both low and excessively high intakes of vitamin D are associated with increased risk of adverse effects and long term outcomes of clinical trials will provide more information on the safety of relatively high levels of 25(OH)D_3_.

## Conclusions

Vitamin D deficiency is still considered a problem of the past by health care professionals and the public. Populations at risk include infants, children, pregnant and postmenopausal women. We have reviewed the existing evidence for a range of possible adverse health outcomes during a women's reproductive period that may relate to low vitamin D status. Besides the classical diseases such as rickets, osteoporosis and osteomalacia, vitamin D deficiency in women might be associated with lower fertility and an increased risk for adverse pregnancy outcomes. Most of the findings in humans are associations or based on animal and laboratory studies and can therefore not determine causality. Available scientific data are limited and well-conducted clinical trials are still lacking. Contradictory results can be explained not only by methodological differences. Genetic, ethnic and racial differences as well as latitude of residence and season may account to the observed discrepancies in several reproductive health outcomes. Vitamin D deficiency is often clinically unrecognized, however laboratory measurements are easy to perform, and treatment of vitamin D deficiency is inexpensive. Oral supplementation is the best-tolerated and the most effective route of administration. At this point optimal levels of 25(OH)D_3 _for the non-classical actions of vitamin D in the reproductive period are not clear. Pressing questions awaiting an answer include the optimal level of vitamin D in women during their reproductive period and especially in pregnancy to achieve maximal benefit for mother and fetus and whether vitamin D supplementation started preconceptionally is protective against preeclampsia and other adverse pregnancy outcomes. High quality, large-scale RCTs are required to determine the optimal 25(OH)D_3 _levels in the reproductive period and the amount of vitamin D supplementation required to achieve those levels for the numerous actions of vitamin D throughout a woman's life. Confirmation of experimental observations relating to the risk of vitamin D deficiency would have important public health implications.

## Abbreviations

CYP27B1: 1α-hydroxylase; DBP: vitamin D-binding protein; GDM: gestational diabetes; IOM: Institute of Medicine; IVF: in vitro-fertilization; PCOS: polycystic ovary syndrome; PTH: parathyroid hormone; PE: preeclampsia; RCT: randomized clinical trial; SPB: spontaneous preterm birth; VDR: vitamin D receptor

## Competing interests

The authors declare that they have no competing interests.

## Authors' contributions

All authors have made substantial contributions to conception and design of the manuscript and have been involved in drafting the manuscript and have given final approval of the version to be published. Both authors read and approved the final manuscript.
